# Lipoblastoma-Related Volvulus in a Nine-Year-Old Female

**DOI:** 10.7759/cureus.62430

**Published:** 2024-06-15

**Authors:** Luke Gingell, Aryana Sharrak, Kristen Hawes, Marc Schlatter

**Affiliations:** 1 Medicine, Michigan State University College of Human Medicine, Grand Rapids, USA; 2 General Surgery, Michigan State University College of Human Medicine, Grand Rapids, USA; 3 Pediatric Surgery, Michigan State University College of Human Medicine, Grand Rapids, USA

**Keywords:** plag-1, pediatric hematology-oncology, upper gastrointestinal surgery, acute volvulus, lipoblastoma

## Abstract

Lipoblastomas are benign neoplasms that arise from embryonal adipocytes. They predominantly impact the pediatric population, with most cases occurring in the first few years of life. These tumors typically present as a soft, palpable, painless mass and tend to involve the mesenchymal tissues of the extremities and trunk. Intraabdominal involvement and intraabdominal complications secondary to lipoblastoma are incredibly rare.

Here, we present the case of a nine-year-old female who presented to the emergency department (ED) with one week of intermittent lower abdominal pain. The CT abdomen/pelvis demonstrated a well-circumscribed hypodense omental mass measuring 10.1 cm x 4.7 cm x 13.4 cm with minimal mass effect or bowel displacement. At that time, the patient’s abdomen was soft without tenderness, distention, or rigidity. Her initial laboratory studies and vital signs were within normal limits. She was evaluated by pediatric surgery, who, given her clinical stability, planned for an anticipated elective resection. Thirteen days after her initial ED visit, the patient returned to the ED with nausea, vomiting, and diffuse abdominal pain. Repeat CT abdomen/pelvis revealed shifting of the omental mass from the left hemi-abdomen to the right hemi-abdomen with associated mesenteric 'swirl sign' and dilated loops of small bowel consistent with small bowel obstruction. Given the patient’s CT findings and signs of peritonitis on a physical exam, she was emergently taken to the operating room, where the mass along with 20 cm of small bowel intimately associated with the mass was resected. The proximal end of the involved bowel was found to be twisted and necrotic, consistent with volvulus. A specimen was sent for cytogenetics and found to be positive for FLAG1, ultimately revealing a diagnosis of lipoblastoma.

The majority of lipoblastoma development is underpinned by gene rearrangements in the zinc-finger transcription factor PLAG1. Although benign, these tumors can exhibit rapid proliferation and have high recurrence rates. Patients should be monitored long-term with ultrasound (US) or MRI following surgery to assess for recurrence.

## Introduction

Lipoblastoma is a rare, benign mesenchymal neoplasm of embryonal white fat that predominantly impacts the pediatric population [1]. Indeed, over 90% of lipoblastoma cases occur during the first three years of life [2,3]. Lipoblastoma has a predilection for male patients, with some studies observing male-female ratios as high as 3:1 [3]. Lipoblastoma typically presents as a soft, palpable, painless mass most commonly involving the superficial connective tissues of the extremities and trunk [2,3]. Approximately 7% of lipoblastomas involve the abdomen [4,5]. However, intraabdominal complications secondary to lipoblastoma are incredibly rare.

## Case presentation

**Figure 1:**
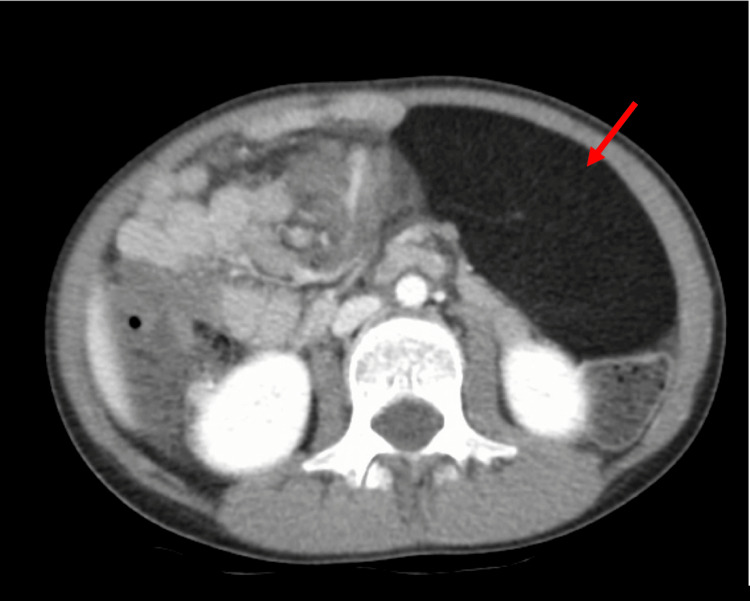
Initial CT scan of the abdomen and pelvis Red arrow: Hypodense omental mass

We report the case of a nine-year-old female who presented to the emergency department (ED) with intermittent lower abdominal pain, dysuria, and urinary frequency for one week. Urinalysis was positive for a small number of leukocytes but negative for nitrites or bacteria. Her initial workup also included an ultrasound (US) of the abdomen/pelvis that showed a large mass in the left upper quadrant of the abdomen. Subsequent CT abdomen/pelvis demonstrated a well-circumscribed hypodense omental mass measuring 10.1 cm x 4.7 cm x 13.4 cm (Figure 1). The CT imaging showed minimal mass effect or bowel displacement. At that time, the patient’s abdomen was soft without tenderness, distention, or rigidity. The patient was evaluated by pediatric surgery, which planned for an anticipated elective resection. Thirteen days after her initial ED visit, the patient returned to the ED with nausea, vomiting, lack of oral intake, and diffuse abdominal pain. 

During the patient’s second ED visit, her vitals included a temperature of 36.6 °C, a heart rate of 109 beats/minute, a respiratory rate of 20 breaths/min, a blood pressure of 109/73, and a peripheral oxygen saturation (SpO2) of 99% on room air. The patient was not in acute distress but appeared uncomfortable. The patient’s weight decreased from 23.6 kg (14th percentile) to 19.7 kg (< 1st percentile) over the past two months. She appeared cachectic. A cardiovascular exam revealed normal S1 and S2 with a regular rate and rhythm. A pulmonary exam showed equal, full-breath sounds bilaterally without wheezing or crackles. Her abdominal exam was notable for significant distention, severe tenderness to palpation, and signs of peritonitis. A complete metabolic panel shows hyponatremia to 125 mmol/L (N:135-145), hypokalemia to 2.4 mmol/L (N:3.5-5), hypochloremia to 79 mmol/L (N:95-110), and hypoglycemia to 49 mg/dl (N:60-100). The patient’s CBC shows a slight decrease in mean corpuscular volume at 78.5 fl but is otherwise within normal limits. 

**Figure 2:**
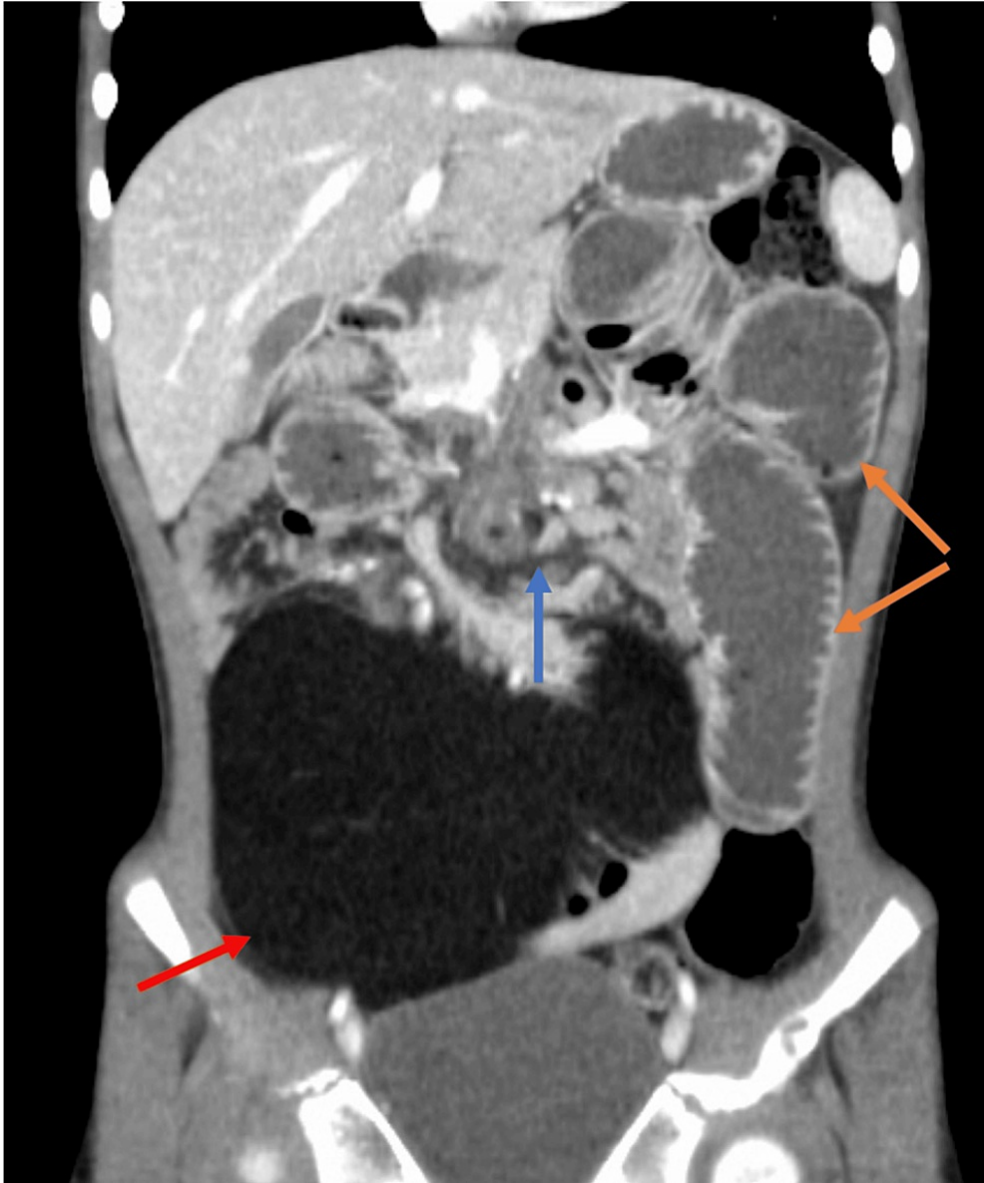
Repeat CT scan of the abdomen and pelvis Red arrow: Hypodense mass, Blue arrow: Mesenteric swirl sign, Yellow arrows: Dilated loops of small bowel

**Figure 3:**
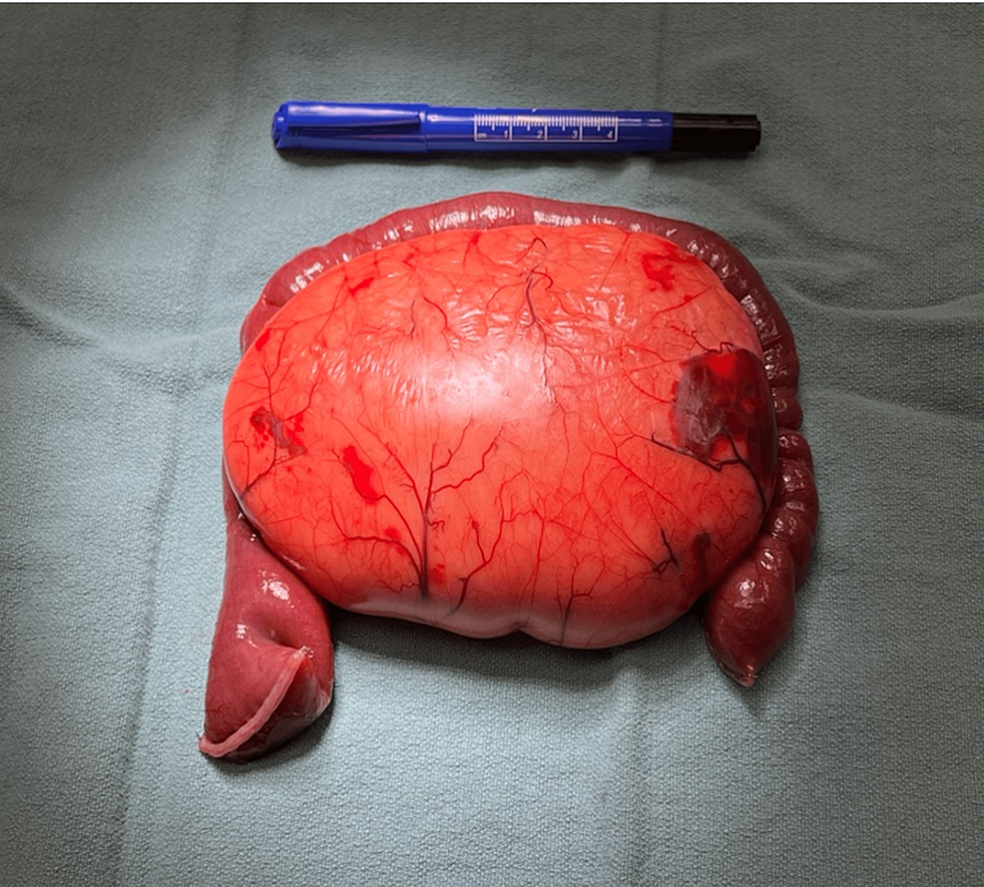
Resected omental mass with associated small bowel

**Figure 4:**
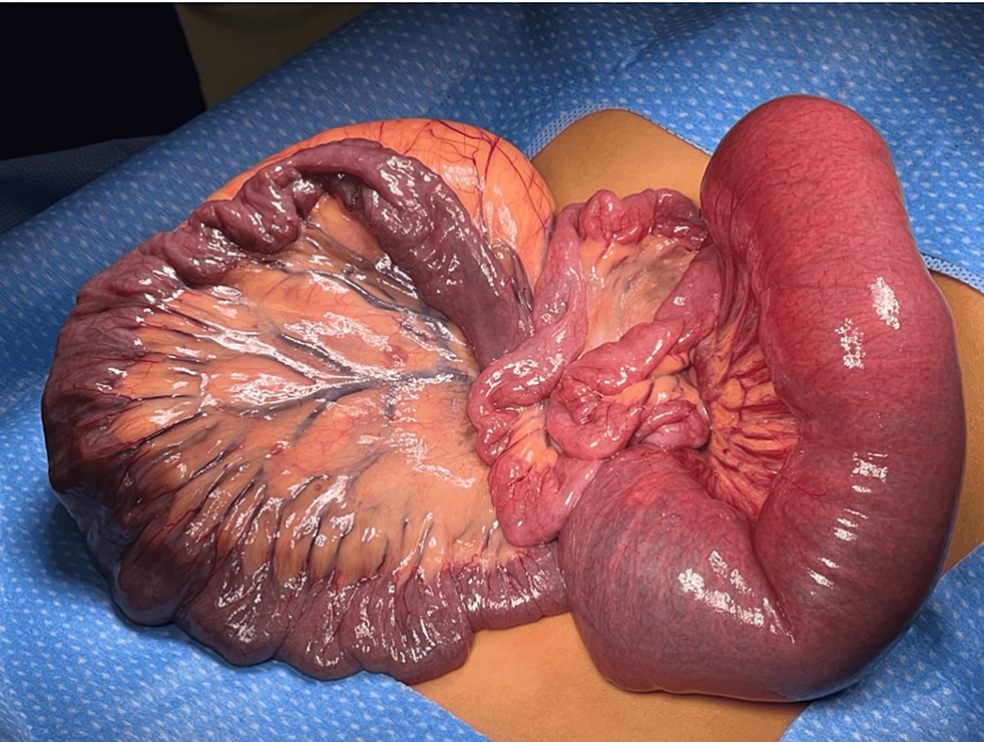
Intraoperative image of twisted and necrotic small bowel

**Figure 5:**
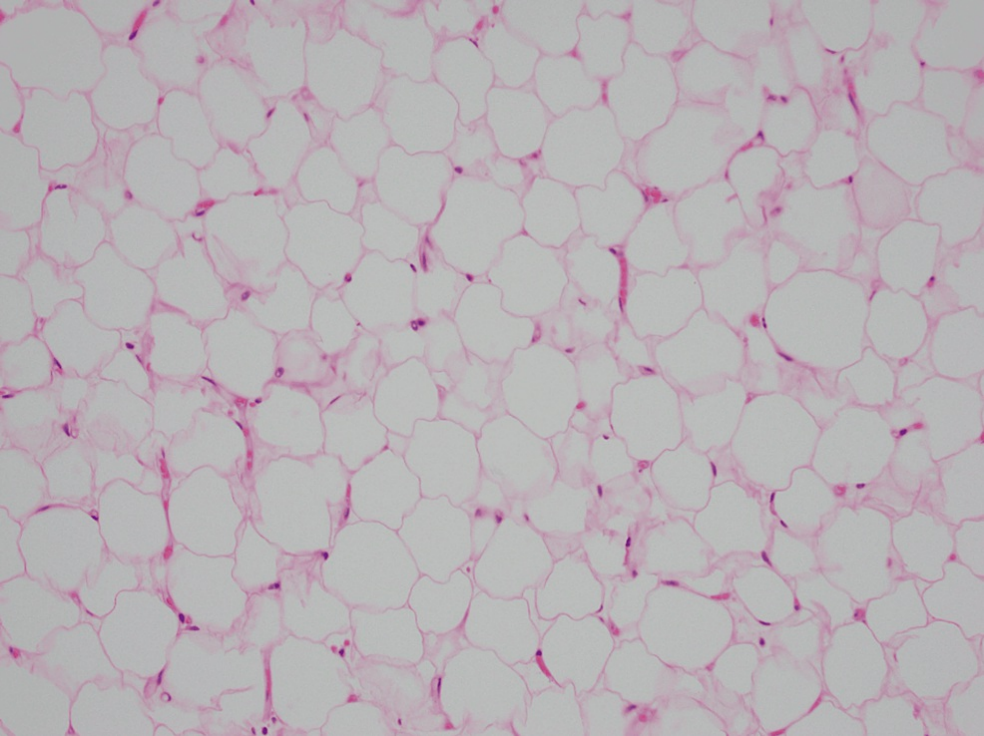
Histologic specimen from the abdominal mass showing mature adipocytes

Repeat CT abdomen/pelvis revealed shifting of the omental mass from the left hemi-abdomen to the right hemi-abdomen with associated mesenteric 'swirl sign' and dilated loops of small bowel consistent with small bowel obstruction (Figure 2). Given the clinical picture with CT findings and peritonitis on exam, the patient was emergently taken to the operating room. A 5 cm midline supraumbilical transverse incision was made, and electrocautery was used to incise the underlying peritoneum. The mass was located within the small bowel mesentery measuring 15 cm x 10 cm. Approximately 20 cm of small bowel was intimately associated with the mass and was not amenable to separation (Figure 3). Due to the likelihood of shared blood supply, we elected to perform a 20 cm small bowel resection with primary anastomosis. The most proximal end of the involved bowel was twisted and necrotic, consistent with volvulus (Figure 4). Histologically, the mass resembled mature adipocytes with vague lobular architecture lacking fibrous septation, zonation, and myxoid change (Figure 5). There was no atypia, multivacuolated lipoblasts, or spectrum of cellular maturation. A specimen was sent for cytogenetics and found to be negative for MDM2 and positive for FLAG1, ultimately revealing a diagnosis of lipoblastoma.

The patient’s mass and involved small bowel were successfully resected with negative margins. Following surgery, the patient had delayed return of bowel function, required total parenteral nutrition (TPN), and developed electrolyte disturbances thought to be associated with re-feeding syndrome (hyponatremia to 127 (N:135-145), hypokalemia to 2.5 (N:3.5-5) with QTc prolongation of 510, and hypophosphatemia to 54 (N:60-100)). She was discharged on post-operation day 6, with a planned follow-up in three to four weeks. The patient will be followed with ultrasounds for surveillance.

## Discussion

The majority of lipoblastoma development is underpinned by gene rearrangements in a region of chromosome 8 encoding the zinc-finger transcription factor PLAG1 [6]. Indeed, 90% of lipoblastoma tumors possess cytogenetic abnormalities in PLAG1, which ultimately leads to the amplification of genes involved in hyaluronic acid and collagen synthesis [2,7-9]. This characteristic genetic aberration helps differentiate lipoblastoma from other adipose tumors such as liposarcoma and is identified by MDM2 amplification, which was negative in our patient [10]. Although benign, these tumors can exhibit rapid proliferation during the postnatal period and have a recurrence rate that approaches 50% [11,12]. This high recurrence rate is thought to result from incomplete resection. Thus, clinical outcomes depend on thorough mass removal and close follow-up after surgery. Patients should be monitored with US or MRI for 10 years following surgery to assess for recurrence [13]. 

## Conclusions

We discussed an atypical presentation of lipoblastoma in a nine-year-old female. Lipoblastoma is most commonly found in male patients less than three years of age. However, our patient did not fall into either of these demographic categories. Thus, providers should consider this rare cause of volvulus in all pediatric patients with a known abdominal mass and signs of bowel obstruction. This case also demonstrates the instability of intraabdominal lipoblastomas, suggesting patients may benefit from more urgent surgical management. Additionally, we reviewed the genetic underpinning and common histologic findings of lipoblastoma. Our work highlights the clinical importance of obtaining clear surgical margins and surveilling lipoblastoma patients for recurrence following surgery.

## References

[1] Kauffman SL, Stout AP (1959 ). Lipoblastic tumors of children. Cancer.

[2] Coffin CM, Alaggio R (2012). Adipose and myxoid tumors of childhood and adolescence. Pediatr Dev Pathol.

[3] Shen LY, Amin SM, Chamlin SL, Mancini AJ (2017). Varied presentations of pediatric lipoblastoma: case series and review of the literature. Pediatr Dermatol.

[4] Squillaro AI, Chow MD, Arias F, Sadimin ET, Lee YH (2020). A giant childhood mesenteric lipoblastoma with extensive maturation. Front Pediatr.

[5] Susam-Sen H, Yalcin B, Kutluk T (2017). Lipoblastoma in children: review of 12 cases. Pediatr Int.

[6] Hibbard MK, Kozakewich HP, Cin PD, Sciot R, Tan X, Xiao S, Fletcher JA (2000). PLAG1 fusion oncogenes in lipoblastoma. Cancer Res.

[7] Bartuma H, Domanski HA, Von Steyern FV, Kullendorff CM, Mandahl N, Mertens F (2008). Cytogenetic and molecular cytogenetic findings in lipoblastoma. Cancer Genet Cytogenet.

[8] Gisselsson D, Hibbard MK, Cin PD, Sciot R, Hsi BL, Kozakewich HP, Fletcher JA (2001). PLAG1 alterations in lipoblastoma: involvement in varied mesenchymal cell types and evidence for alternative oncogenic mechanisms. Am J Pathol.

[9] Yoshida H, Miyachi M, Ouchi K (2014). Identification of COL3A1 and RAB2A as novel translocation partner genes of PLAG1 in lipoblastoma. Genes Chromosomes Cancer.

[10] Sciot R (2021). MDM2 amplified sarcomas: a literature review. Diagnostics (Basel).

[11] Coffin CM, Lowichik A, Putnam A (2009). Lipoblastoma (LPB): a clinicopathologic and immunohistochemical analysis of 59 cases. Am J Surg Pathol.

[12] Chun YS, Kim WK, Park KW, Lee SC, Jung SE (2001). Lipoblastoma. J Pediatr Surg.

[13] Séguier-Lipszyc E, Baazov A, Fichman S, Ash S, Freud E (2018). Current management of lipoblastoma. Eur J Pediatr.

